# Can Ag^+^ Permeate through a Potassium Ion Channel? A Bottom-Up Approach by Infrared Spectroscopy of the Ag^+^ Complex with the Partial Peptide of a Selectivity Filter

**DOI:** 10.1021/acs.jpclett.2c03366

**Published:** 2023-03-16

**Authors:** Satoru Tanabe, Keisuke Hirata, Koichi Tsukiyama, James M. Lisy, Shun-ichi Ishiuchi, Masaaki Fujii

**Affiliations:** † Department of Chemistry, School of Science, Tokyo University of Science, 1-3 Kagurazaka, Shinjuku-ku, Tokyo 162-8601, Japan; ‡ Laboratory for Chemistry and Life Science, Institute of innovative research, Tokyo Institute of Technology, 4259 Nagatsuta-cho, Midori-ku, Yokohama 226-8503, Japan; § Department of Chemistry, School of Science, Tokyo Institute of Technology, 2-12-1 Ookayama, Meguro-ku, Tokyo 152-8550, Japan; ∥ International Research Frontiers Initiative (IRFI), Institute of Innovation Research, Tokyo Institute of Technology, 4259, Nagatsuta-cho, Midori-ku, Yokohama 226-8503, Japan; ⊥ Department of Chemistry, University of Illinois at Urbana—Champaign, Urbana, Illinois 61801, United States; # School of Life Science and Technology, Tokyo Institute of Technology, 4259 Nagatsuta-cho, Midori-ku, Yokohama 226-8503, Japan

## Abstract

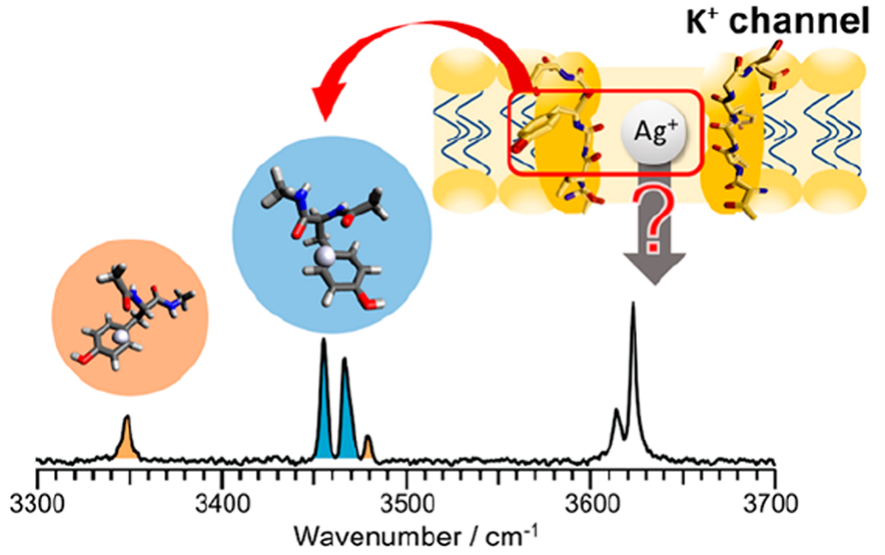

Silver and silver ions have a long history of antimicrobial activity and medical applications. Nevertheless, the activity of Ag^+^ against bacteria, how it enters a cell, has not yet been established. The K^+^ channel, a membrane protein, is a possible route. The addition of a channel inhibitor (4-aminopyridine) to modulate the Ag^+^ uptake could support this view. However, the inhibitor enhances the uptake of Ag^+^, the opposite result. We have applied cold ion trap infrared laser spectroscopy to complexes of Ag^+^ and Ac-Tyr-NHMe (a model for GYG) which is a portion of the selectivity filter in the K^+^ channel to consider the question of permeation. With support from quantum chemical calculations, we have determined the stable conformations of the complex. The conformations strongly suggest that Ag^+^ would not readily permeate the K^+^ channel. The mechanism of the unexpected enhancement by the inhibitor is discussed.

It is well-known that silver(I) and silver nanoparticles have antimicrobial activity^1−7^ and have been applied to burn remedies,^8,9^ dental care,^10,11^ and the material used in medical instruments^12,13^ as examples. Despite these wide applications, the antimicrobial mechanism of silver ions has not been established. So far, two mechanisms have been proposed: (1) Ag^+^ lyses cells by interacting with cell walls and membranes and (2) Ag^+^ inhibits DNA replication and protein synthesis.^1,2,14^ In the latter mechanism, Ag^+^ must operate inside the cells and is thought to be transported through membrane proteins such as ion channels.^14^ The K^+^ channel is one possible membrane protein, as Ag^+^ uptake is modulated by adding the K^+^ channel inhibitor, 4-aminopyridine (4-AP).^15^ While providing evidence that the K^+^ channel plays a role in the uptake of Ag^+^, the action of 4-AP is opposite to what was expected; it enhances Ag^+^ uptake, even though 4-AP is a channel inhibitor. Therefore, further study is necessary to clarify whether Ag^+^ permeates via K^+^ channels or not.^15^

K^+^ channels are membrane proteins that selectively transmit K^+^, and not ions with smaller ionic radii such as Li^+^, Na^+^, or divalent alkaline earth metal ions. The structure of these channels and the mechanism of ion selectivity have been studied extensively. The crystal structure of one such channel, KcsA, determined by MacKinnon and co-workers,^16^ was a major breakthrough in the field. They found that the ion selectivity is achieved by the interactions of K^+^ with the amino acid sequence Thr-Val-Gly-Tyr-Gly (TVGYG), which forms (with three more identical units) the selectivity filter (SF).^17^ The K^+^ interacts with eight carbonyl oxygens in the SF with a coordination similar to the hydration shell of K^+^ and with comparable energetics. The coordination by the carbonyl oxygens effectively compensates for the hydration energy of K^+^, allowing the ion to diffuse from water into the channel with only a small energetic cost. In contrast, Na^+^, which is smaller than K^+^, does not fit as well in the SF as K^+^. The change in configuration imposes a large energetic barrier for the permeation of Na^+^. This interpretation suggests that the ionic radius of the metal ion is one key factor in permeation through the K^+^ channel. As shown in Table 1, the ionic radius of Ag^+^ is larger than Na^+^ and relatively close to K^+^.^18^ It suggests permeation of Ag^+^ through a K^+^ channel may be possible; however, to the best of our knowledge, no molecular-level studies, such as X-ray diffraction or molecular dynamics simulation have addressed this question of Ag^+^ permeability.

**Table 1:** Ionic Radii^a^ of K^+^, Ag^+^, and Na^+^

	K^+^	Ag^+^	Na^+^
ionic radius/Å	1.33	1.26	0.95

a Pauling ionic radius.

We have found that the permeability of alkali and alkaline earth metal ions for the K^+^ channel well correlates to the conformation of a partial peptide of SF, Ac-Tyr-NHMe (our model for GYG, with methylation of the C and N termini), when Ac-Tyr-NHMe (hereafter called GYG) binds the metal ion.^19−21^ GYG prefers the bidentate structure in which the metal ion is coordinated with the two C=O groups (named as O/O, O/O′, Figure 1) when complexed with K^+^, a permeable ion. This structure is similar to that found in the SF. On the other hand, the impermeable ions, such as Na^+^, favor coordination with GYG in a tridentate conformation (O/O/R, Figure 1) in which the metal ion is secured by the two C=O groups and the aromatic side chain of Tyr. This additional interaction cannot be achieved in actual K^+^ channels, which implies that the ion (Na^+^) does not coordinate with the SF to facilitate permeation.

**Figure 1:**
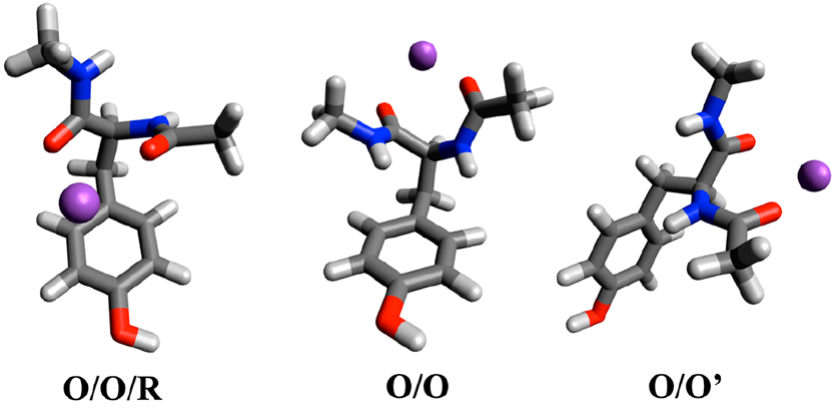
GYG-K^+^ conformer structures: tridentate (O/O/R) and bidentate (O/O, O/O′). The three conformers for all ionic species are shown in Figure S1.

Based on these correlations, we attempt to examine the possibility of Ag^+^ permeation through the K^+^ channel by measuring the infrared (IR) spectra of the GYG-Ag^+^ complex to determine its conformation. The GYG-Ag^+^ complex is introduced into a vacuum by an electrospray ionization source and isolated in a cryogenic ion trap after mass selection.^22−26^ The IR spectra are measured via IR photodissociation (IRPD) spectroscopy with the H_2_ tagging method.^27^

Figure 2a shows the IR spectra of the GYG-Ag^+^ complex. For comparison, the spectra of GYG-K^+^, GYG-Na^+^, and GYG-Ca^2+^ are also shown in Figure 2b–d.^19,20^ Vibrational bands in 3300–3500 and 3630–3650 cm^–1^ are assigned to NH and OH stretches, respectively. In this spectral range, the NH stretching bands (amide A) are marker bands that are used to identify the conformation of the GYG peptide. For GYG-K^+^, the six NH bands are assigned to two bidentate conformers (O/O and O/O′ indicated by green and red, respectively) and one tridentate conformer (O/O/R indicated by blue). As each peptide has two N–H groups the six NH stretching bands demonstrate the coexistence of three separate conformers. These same conformers are also found in the IR spectrum of GYG-Na^+^. Population estimates of conformers can be obtained from the intensities of the bands and the NH stretch oscillator strengths. From the analysis, previously published, the O/O and O/O′ bidentate conformations are dominant in GYG-K^+^, while the tridentate conformer is preferred in GYG-Na^+^, showing the difference in binding conformations for these two ions. In the case of the Ag^+^ complex, four NH stretching bands (3479, 3467, 3455, and 3348 cm^–1^) are observed in the amide A range, from which one can infer that GYG-Ag^+^ has only two conformers, with the two bands at 3467 and 3455 cm^–1^ dominating the spectrum. This spectral feature is reminiscent of the IR spectra of the GYG-M^2+^ complexes with alkaline earths, such as Ca^2+^ (see Figure 2d). The intense doublet in the IR spectra of the GYG-M^2+^ complexes was assigned to the NH stretching bands of the O/O/R conformers, and we tentatively assign that the two bands in Figure 2a at 3467 and 3455 cm^–1^ (as supported by theoretical calculations, vide infra) to the two NH stretch modes of the O/O/R conformation. The other two bands at 3479 and 3348 cm^–1^ are assigned to NH stretching bands of a second minor conformer, not previously observed for the alkali and alkaline earth ion-GYG complexes. The two bands in the OH stretching region also support the existence of a second conformer. Additional spectra in the 6 μm region of the C=O stretch are presented in the Supporting Information, Figure S2, for all four ions.

**Figure 2:**
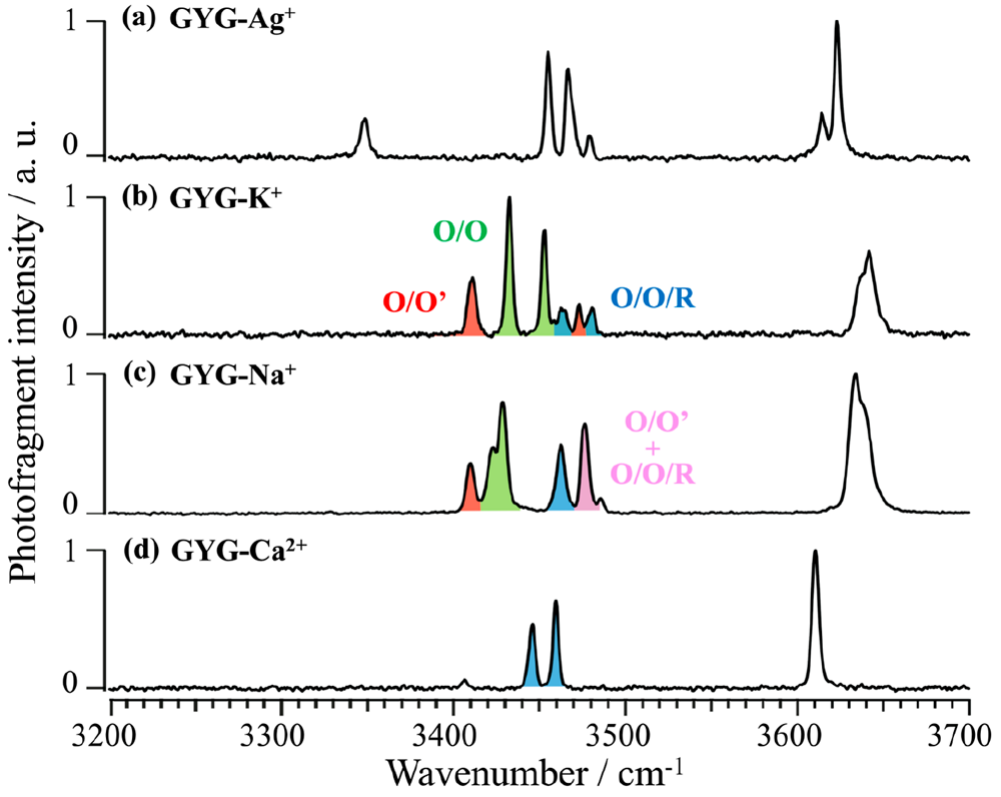
IRPD spectrum of (a) GYG-Ag^+^, (b) GYG-K^+^, (c) GYG-Na^+^, and (d) GYG-Ca^2+^. Spectra shown in panels b–d are adapted with permission from refs (19) and (20). Copyrights 2019 Royal Society of Chemistry and 2020 Wiley. Spectral assignments for the amide A range are color-coded: green for O/O, red for O/O′, blue for O/O/R, and purple for O/O′ and O/O/R.

To establish the assignments of conformers, quantum chemical calculations at the functional B3LYP-D3 level and the basis sets cc-pVTZ (for C, N, O, H) and SDD (Ag) levels were applied to the GYG-Ag^+^ complex. Previously reported conformations and newly generated conformers, by rotating the dihedral angles (χ_1_, φ, and ψ)^28^ of the GYG peptide, are used as initial geometries of calculations (see Figure S3) in Supporting Information, and 11 optimized structures are obtained as shown in Figure S4 in the Supporting Information. The computed IR spectra for the four most stable conformers (O/O/R, O/R, O/O, and O/O′ conformers) are shown in Figure 3, together with the observed spectrum. A comparison between the theoretical and experimental IR spectra are displayed in Figure S5. The most stable conformer in terms of the Gibbs free energy (Δ*G*) at 298 K is the tridentate O/O/R, in which the metal ion is coordinated by two carbonyl groups and the phenyl ring of tyrosine. Comparing the measured and calculated spectra, the observed intense doublet (3467, 3455 cm^–1^) is assigned to calculated NH bands of O/O/R (3464 cm^–1^ for NH(B), 3459 cm^–1^ for NH(A)).^28^ Here, notations of NH(B) and NH(A) are defined in the Supporting Information (Figure S3e), where NH(A) is at the acetyl end of the peptide. The prominent band at 3623 cm^–1^ is assigned to the Tyr-OH stretch of O/O/R. The second most stable structure is the bidentate O/R in which the metal ion is coordinated to a single carbonyl group (acetyl C=O) and the phenyl ring of tyrosine. The bands at 3479 and 3348 cm^–1^ are assigned to the NH stretching bands of the O/R (3467 cm^–1^ calculated for NH(B), 3360 cm^–1^ for NH(A)). A large red shift of the NH(A) stretch is caused by an intramolecular hydrogen bond NH···OC (see Figure 3). The shoulder at 3614 cm^–1^ is assigned to the Tyr-OH stretch of the O/R conformer. The coexistence of O/O/R and O/R is also supported by vibrational signatures of the CO stretch in the 6 μm range (Figure S5). While the O/O/R conformation is found in other GYG complexes with alkali and alkaline earth metal ions, the O/R conformation of GYG-Ag^+^ is unique in bare GYG-metal ion complexes. It suggests a substantial attraction between Ag^+^ and the aromatic ring. This likely arises from significant interactions between 4d electrons and 5s electrons in Ag^+^ and the π ring.^29−32^ To evaluate the extent of these interactions, the M^+^···C=C interaction energies between the Tyr-ring and the three metal ions, K^+^, Na^+^, and Ag^+^, in the O/O/R conformer were determined by natural bond orbital (NBO) analysis, as given in Table 2. Here, the interaction energy *E(1)* represents the extent of σ-donation from the π electron of C=C to the empty s orbital of the metal ion. *E(2)* indicates the extent of π-back-donation from the p orbital or d orbital electron of the metal ion to the antibonding orbital π* of C=C. For the Ag^+^ complex, *E(1)* of the C=C(π) → Ag^+^(5s) orbital interaction was estimated to be 10.54 kcal/mol. And *E(2)* of the Ag^+^(4d) → C=C(π*) interaction was estimated to be 4.35 kcal/mol. In comparison, the values for *E(1)* for Na^+^ and K^+^ [C=C(π) → Na^+^(3s), 1.29 kcal/mol; C=C(π) → K^+^(4s), 0.21 kcal/mol] and *E(2)* for Na^+^ and K^+^ [Na^+^(2p) → C=C(π*), 0.12 kcal/mol; K^+^(3p) → C=C(π*), 0.07 kcal/mol] are much smaller. The *E(1)* and *E(2)* values for the Ag^+^ complex differ from those of other metal ion complexes by at least a factor of 10. This indicates the interaction between Ag^+^ and the Tyr-ring is strong, suggesting some degree of covalency (coordinate covalent bond). The bidentate O/O and O/O′ conformers, in which two carbonyl groups coordinate to the metal ion, are less stable than O/O/R and O/R, lacking the stabilization from the strong interaction with the aromatic ring. Indeed, we could not find any band which is assignable to the NH stretching in the higher energy O/O or O/O′ conformers which contrasts with their dominance in permeable ions such as K^+^. As well as O/O/R, the O/R conformation is far from a feasible configuration that would enable Ag^+^ to permeate the K^+^ channel. These results suggests that Ag^+^ should be grouped with Li^+^, Na^+^, and alkaline earth metal ions such as Ca^2+^ that do not permeate through K^+^ channels. While Ag^+^ has a similar ion radius to K^+^, our analysis suggests that it is not a key factor for the permeation of the K^+^ channel.

**Table 2:** M^+^–π Interaction Energy Obtained by NBO Analysis^a^

Metal ion	C=C(π) → M^+^(s) interaction energy *E*(1)/kcal mol ^–1^	M^+^(d/p)→ C=C(π*) interaction energy *E*(2)/kcal mol ^–1^
Ag^+^	10.54	4.35
K^+^	0.21	0.07
Na^+^	1.29	0.12

a More details for NBO analysis are mentioned in the Supporting Information.

**Figure 3:**
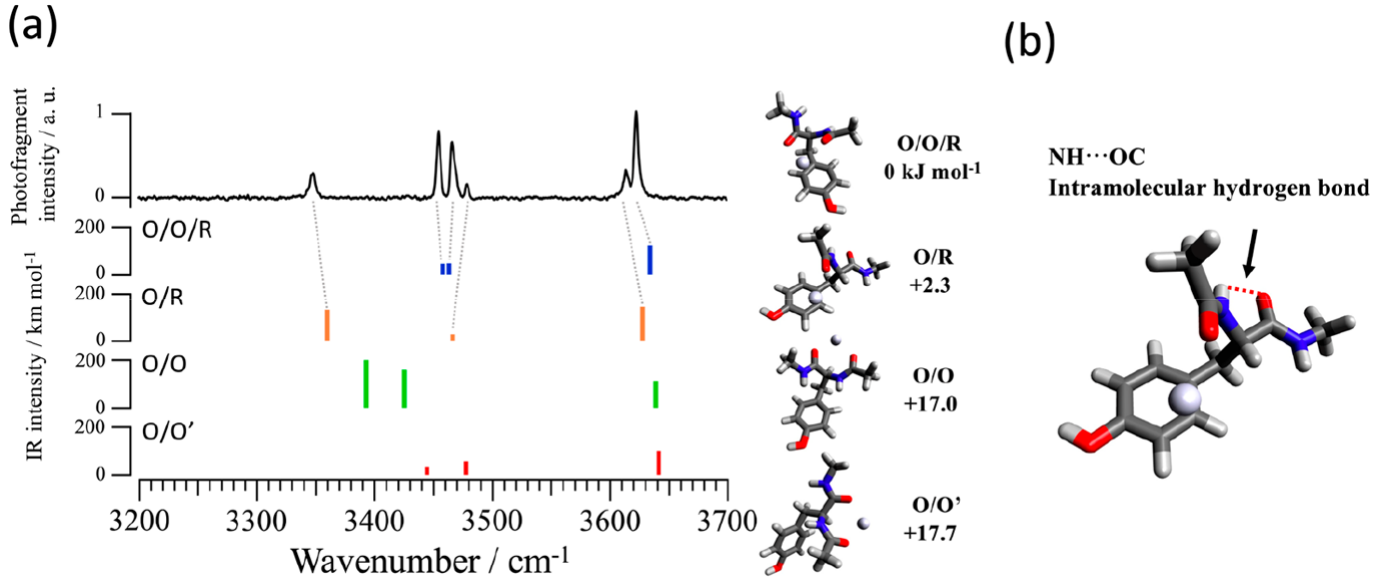
(a) IRPD spectra of GYG-Ag^+^ compared with theoretical spectra at the B3LYP-D3/cc-pVTZ (other atoms) and SDD (Ag) (scaling factor: 0.956 (amide A)) levels. Each structure and Gibbs free energy Δ*G*(298 K) corresponding to the calculation are shown next to the spectra. The NH–OC hydrogen bond is shown in the enlarged O/R structure in (b).

This conclusion is also confirmed by considering the effect of temperature on the relative population of the conformers. The conformer distributions are affected by temperature because more flexible conformers with higher entropy can be favored at higher temperatures. For the GYG complex with permeable ions (K^+^, Rb^+^ and Cs^+^), population of O/O and O/O′ conformations (feasible ones for the channel permeation) increase with increasing temperature and become dominant at near physiological temperatures (300 K).^21^ On the other hand, nonfeasible enthalpically favored O/O/R conformers are dominant at all temperatures when GYG forms a complex with nonpermeable Li^+^/Na^+^ ions. For the GYG-Ag^+^ complex, the nonfeasible O/O/R and O/R conformations are dominant at all temperatures as well (see Figure S6 in the Supporting Information). Rigid enthalpically favored ion-GYG complexes lack the flexibility to lead to efficient permeation.

Our results suggest that Ag^+^ does not permeate through the K^+^ channel. Then, how can one explain the enhancement of Ag^+^ uptake in cells when the K^+^ channel blocker, 4-AP, is present? Na^+^ channels, also present in the cells, may take on a significant role for this unexpected enhancement. It is proposed that Ag^+^ may permeate through the voltage-dependent Na^+^ channel, since the presence of Na^+^ channel blockers (Phenamil and bafilomycin A_1_)^33^ prevents the uptake of Ag^+^. As 4-AP does not block the Na^+^ channel, Ag^+^ would be taken up via the Na^+^ channel. When the membrane potential exceeds its threshold, the Na^+^ channel is opened,^34^ and both Na^+^ and Ag^+^ can enter the inner cell. The Na^+^ channel remains open until the membrane potential returns to normal, which is achieved by the release of K^+^ through the voltage-dependent K^+^ channel. Because 4-AP blocks the K^+^ channels, the Na^+^ channels are expected to remain open for a longer period. Thus, it is expected that 4-AP can promote the uptake of Ag^+^ through the open Na^+^ channel proteins.

## Experimental Section

Figure S7 in the Supporting Information depicts the experimental setup, and details can be found in our previous study.^23^ To prepare the metal complexes, Ac-Tyr-NHMe (2 × 10^–4^ M, purchased from BACHEM) and AgNO_3_ (2 × 10^–4^ M, 99.8%, purchased from Wako Ikkyu Co.) were dissolved in pure methanol. This sample was electrosprayed from an emitter to produce fine droplets containing the complex ions. The droplets were desolvated via a glass capillary heated to 80 °C and introduced into a vacuum. The resulting complex ions were collected by an ion funnel and introduced into a first quadrupole mass spectrometer (Q-MS) with a hexapole ion guide. The target species GYG-Ag^+^ was mass-selected by the Q-MS1. In measuring the temperature dependence, mass-selected GYG-Ag^+^ complexes were guided into a temperature-controlled octupole linear ion trap (at selected temperatures between 180 to 300 K) and trapped for 50 ms, while colliding with He buffer gas. Then GYG-Ag^+^ was introduced into a quadrupole ion trap (QIT) cooled to 4 K by a closed cycle He refrigerator via Q-MS2 and a hexapole ion guide. H_2_ (20%)/He buffer gas was injected into the QIT via a pulse valve. During the 40 ms trapping period, the sample ions were cooled to ∼10 K by collisions with the buffer gas mixture that also enabled H_2_ molecules to be attached to the ions. The generated and trapped H_2_-tagged GYG-Ag^+^·H_2_ ions were then irradiated with a tunable infrared laser (LaserVision, OPO/OPA). When vibrational excitation is induced by the absorption of a resonant infrared laser photon, the H_2_-dissociated fragment GYG-Ag^+^ was produced and detected by a time-of-flight mass spectrometer (TOF-MS). Infrared photodissociation (IRPD) spectra were measured by recording the signal intensity of this fragment as a function of IR wavenumber.

To obtain the optimized structure of the complex and the corresponding IR spectra, density functional theory (DFT) calculations at the functional B3LYP-D3 level and the basis sets cc-pVTZ (for C, N, O, H) and SDD (Ag) levels were performed using the Gaussian16 package. The harmonic frequencies were scaled by a linear scaling factor of 0.956 in the OH and NH stretching regions and 0.975 in the other regions.^19^

## Supporting Information Available

Calculated structures, IRPD spectra including the 3 μm + 6 μm region, initial and optimized structures of GYG-Ag^+^, calculated distances, theoretical spectra of all optimized structures, vibrational frequencies, the experimental setup, and natural bond orbital (NBO) analysis results, and structural coordinate data (PDF)
