# Supplementary material for: Can Ag+ Permeate through a Potassium Ion Channel? A Bottom-Up Approach by Infrared Spectroscopy of the Ag+ Complex with the Partial Peptide of a Selectivity Filter
Source: J Phys Chem Lett. 2023 Mar 16;14(11):2886–90. doi: 10.1021/acs.jpclett.2c03366 (PMC10041629; doi:10.1021/acs.jpclett.2c03366)
Supplement: Supplementary file 1 — jz2c03366_si_001.pdf [file jz2c03366_si_001.pdf]

Supporting Information

Can Ag^+ Permeate Through a Potassium Ion Channel? – A Bottom-Up Approach by Infrared Spectroscopy of the Ag^+ Complex with the Partial Peptide of a Selectivity Filter

Satoru Tanabe[1,2], Keisuke Hirata[2,3,4], Koichi Tsukiyama[1], James M. Lisy*[4,5], Shun-ichi Ishiuchi*[2,3,4], and Masaaki Fujii*[2,4,6]

[1] *Department of Chemistry, School of Science, Tokyo University of Science, 1-3 Kagurazaka, Shinjuku-ku, Tokyo, 162-8601, Japan*

[2] *Laboratory for Chemistry and Life Science, Institute of innovative research, Tokyo Institute of Technology, 4259 Nagatsuta-cho, Midori-ku, Yokohama, 226-8503, Japan*

[3] *Department of Chemistry, School of Science, Tokyo Institute of Technology, 2-12-1 Ookayama, Meguro-ku, Tokyo, 152-8550, Japan*

[4] *International Research Frontiers Initiative (IRFI), Institute of Innovation Research, Tokyo Institute of Technology, 4259, Nagatsuta-cho, Midori-ku, Yokohama, 226-8503 Japan*

[5] *Department of Chemistry, University of Illinois at Urbana-Champaign, Urbana, IL 61801, USA*

[6] *School of Life Science and Technology, Tokyo Institute of Technology, 4259 Nagatsuta-cho, Midori-ku, Yokohama, 226-8503, Japan*

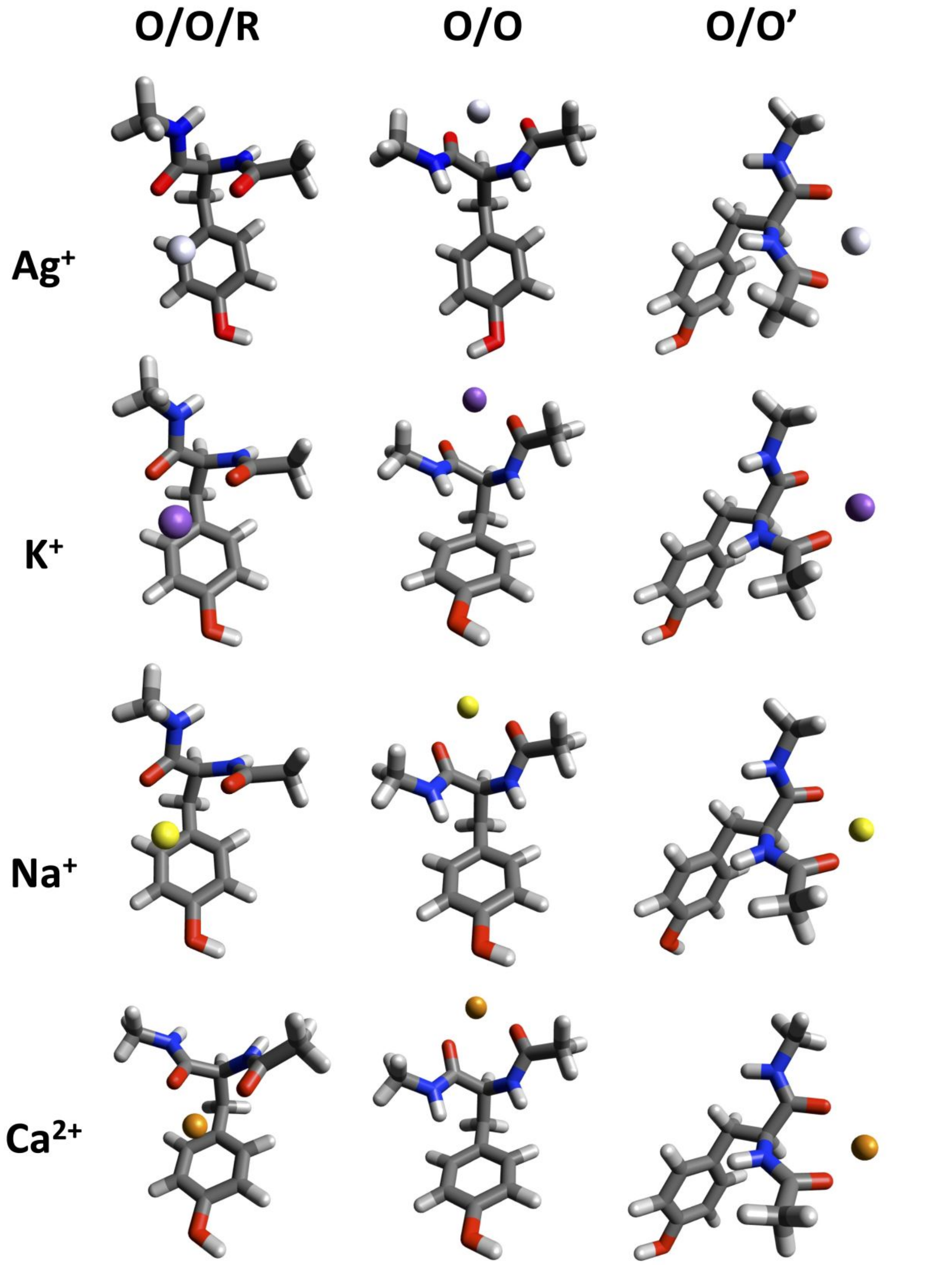

Fig. S1 Calculated structures of GYG-M$^+$(M$^+$=Ag$^+$, K$^+$, Na$^+$, Ca$^+$). Each isomer has rotamer of the phenolic OH and more stable one is shown. Conformer, O/O/R, O/O and O/O' are shown.

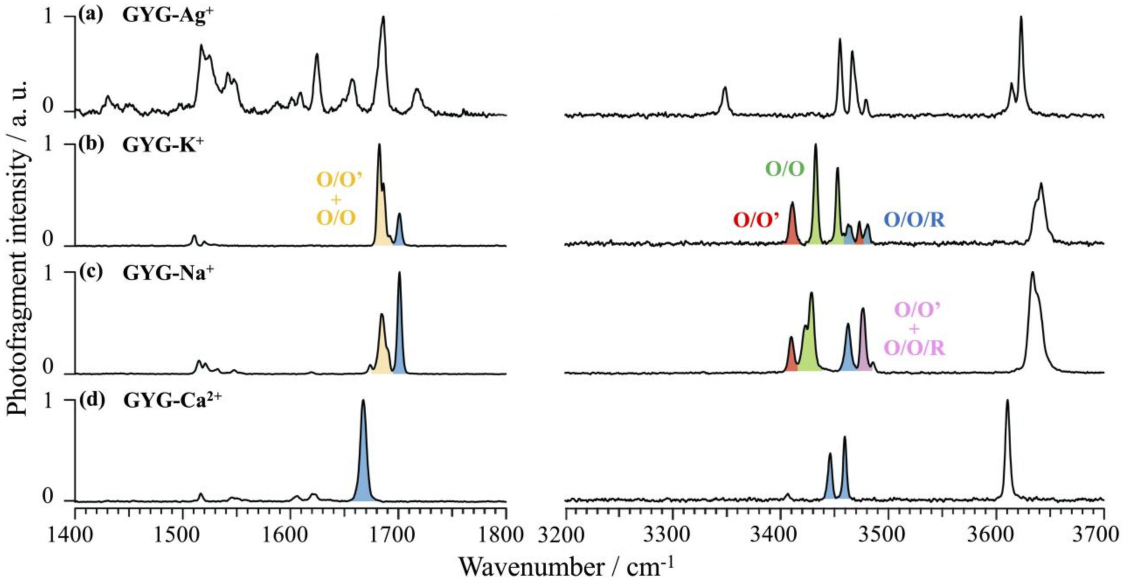

Fig. S2 IRPD spectrum 3 μm + 6 μm region of (a) GYG-Ag^+, (b) GYG-K^+, (c) GYG-Na^+, and (d) GYG-Ca^{2+}. Spectra shown in Fig. 2b-2d are adapted from refs[1, 2]. Spectral assignments for the amide A range are color-coded: green for O/O, red for O/O', blue for O/O/R, and purple for O/O' and O/O/R.

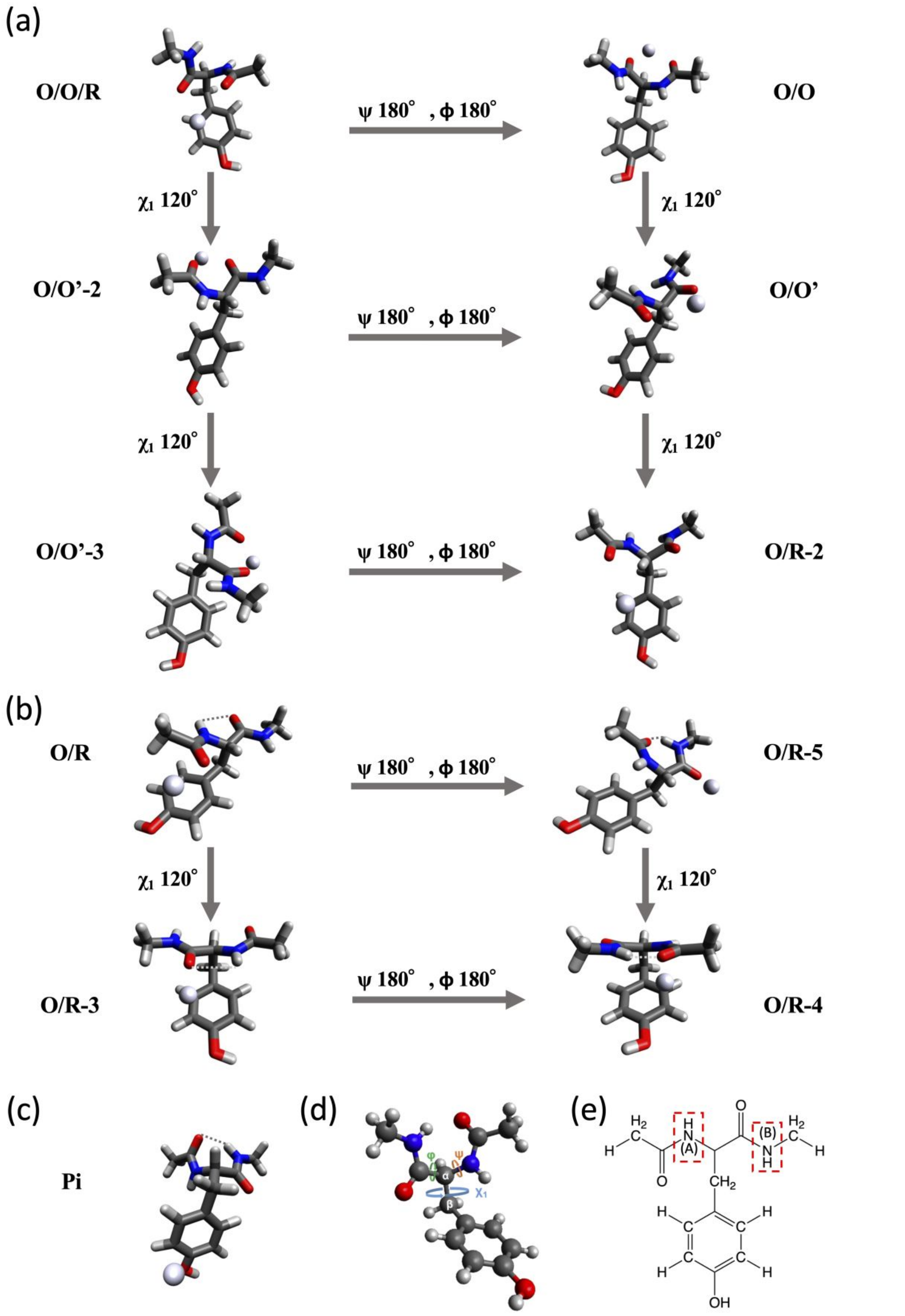

Fig. S3 Initial structures of GYG-Ag^{+}. (a) O/O/R series, (b) O/R series, (c) Pi series, (d) definition of the dihedral angle with respect to the backbone and side chain of GYG and (e) definition of NH(A) and NH(B) notation respect to the NH groups of GYG. Indicated angle is used for making initial structures.

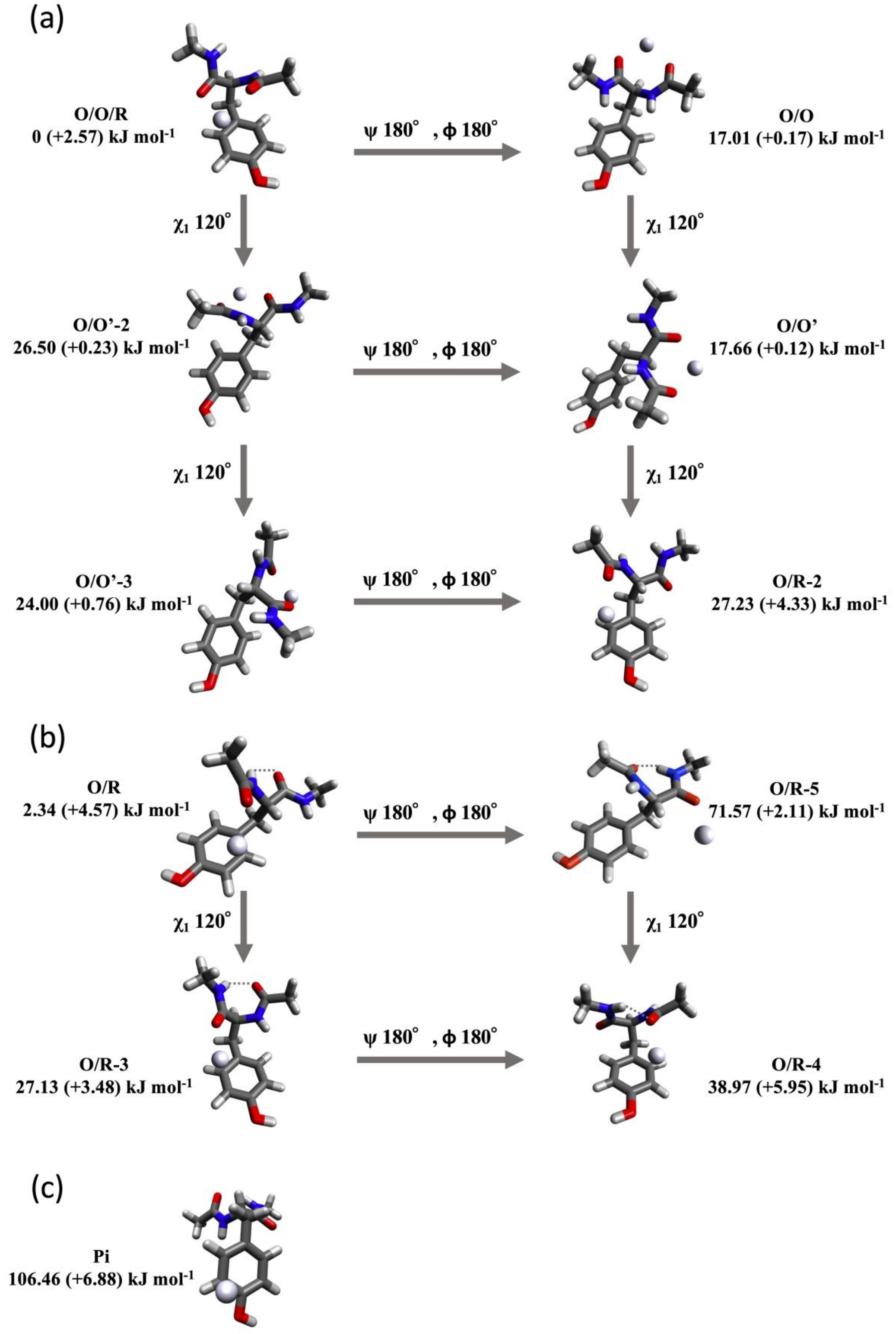

Fig. S4 Calculated structures of GYG-Ag^+ with Gibbs free energy at 298 K. Each isomer has rotamer of the phenolic OH and more stable one is shown. (a) O/O/R series, (b) O/R series, (c) Pi series. Numbers in parentheses indicate free energy difference of Tyr-OH rotamer. Calculated distances (Å) for Ag^+, oxygen, nitrogen, hydrogen, and C=C for the most stable structure of each O/O/R, O/R, O/O and O/O' conformers, are summarized in the Table S1.

Table S1. Calculated distances (Å) for Ag^+, oxygen, nitrogen, hydrogen, and C=C. Values are presented for the more stable Tyr-OH rotamer of each conformer.

	O/O/R	O/R	O/O	O/O'
Ag^+···O(Ac)	2.35	2.18	2.27	2.27
Ag^+···O(NHMe)	2.47	–	2.26	2.26
O···O	3.17	–	3.47	3.47
(Ac)O···HN(B)	–	–	–	–
(NHMe)O···HN(A)	–	2.14	–	–
(N)H···H(N)	3.22	–	3.01	3.41
N(A)-H···π	–	–	3.33	3.78
N(B)-H···π	–	–	2.93	5.43
Ag^+···C=C	2.46	2.34	–	–

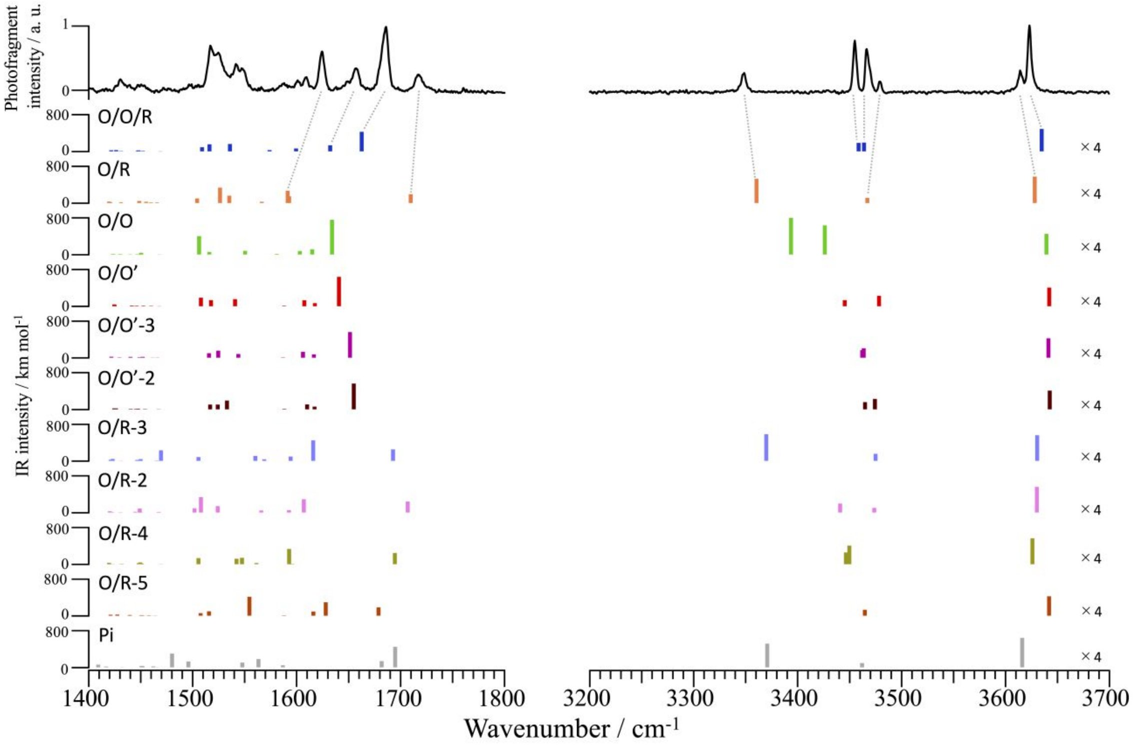

Fig. S5 IRPD spectra of GYG-Ag$^+$ compared with calculated spectra at B3LYP-D3/cc-pVTZ (C, N, O, H), SDD (Ag) (scaling factor: 0.956(Amide A), 0.975(Amide Ⅰ, Ⅱ)). Calculated IR intensities for 3 μm region are enhanced by a factor of 4. Wavenumber values for the specific vibrations are summarized in the Table S2.

Table S2. Observed and calculated vibrational frequencies (cm^{-1}) and their assignments.

obs.					calc.							Isomer assignment
OH str.	NH str. (A)	NH str. (B)	CO str. (sym)	CO str. (anti)	OH str.	NH str. (A)	NH str. (B)	NH str. (sym)	NH str. (anti)	CO str. (sym)	CO str. (anti)	
3623	3455	3467	1687	1657	3635	3459	3464	–	–	1662	1632	O/O/R
3614	3348	3479	1717	1625	3628	3360	3467	–	–	1710	1593 / 1591	O/R
					3639	3426	3394	–	–	1634	1603	O/O
					3642	3445	3478	–	–	1641	1608	O/O'
					3641	3462	3464	–	–	1652	1606	O/O'-3
					3642	3465	3474	–	–	1655	1610	O/O'-2
					3631	3475	3370	–	–	1616	1693	O/R-3
					3630	3441	3474	–	–	1707	1607	O/R-2
					3626	–	–	3446	3450	1592	1695	O/R-4
					3642	3465	3110	–	–	1554	1679	O/R-5
					3621	3464	3368	–	–	1682	1695	Pi

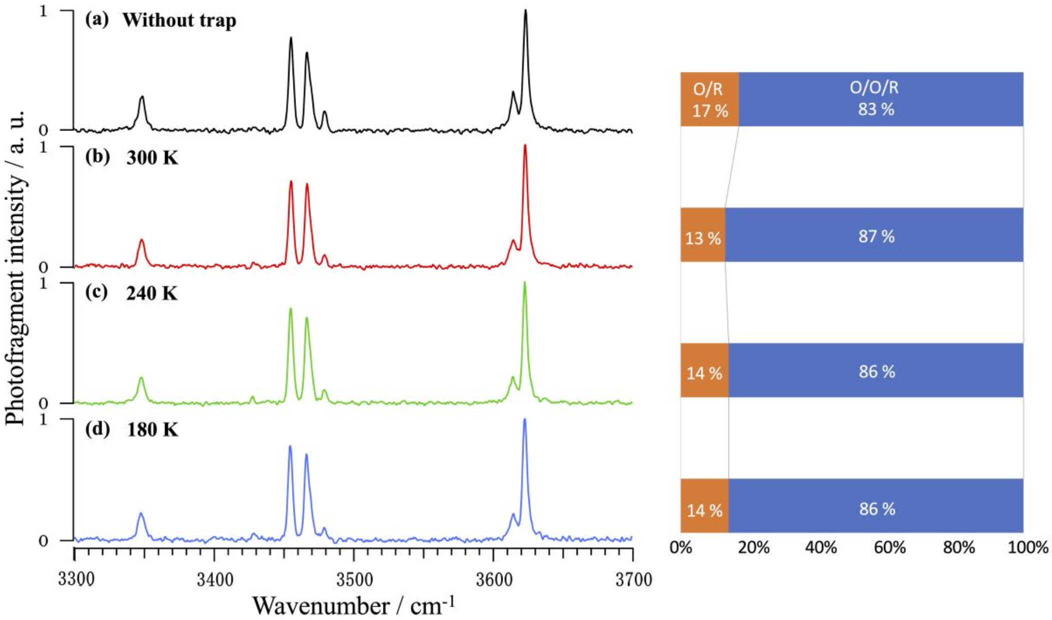

Fig. S6 IRPD spectra at given temperatures (a) without trap, (b) 300, (c) 240, (d) 180 K of GYG-Ag^+ complex and abundance ratio. O/O and O/O' are not observed and not listed in bar chart.

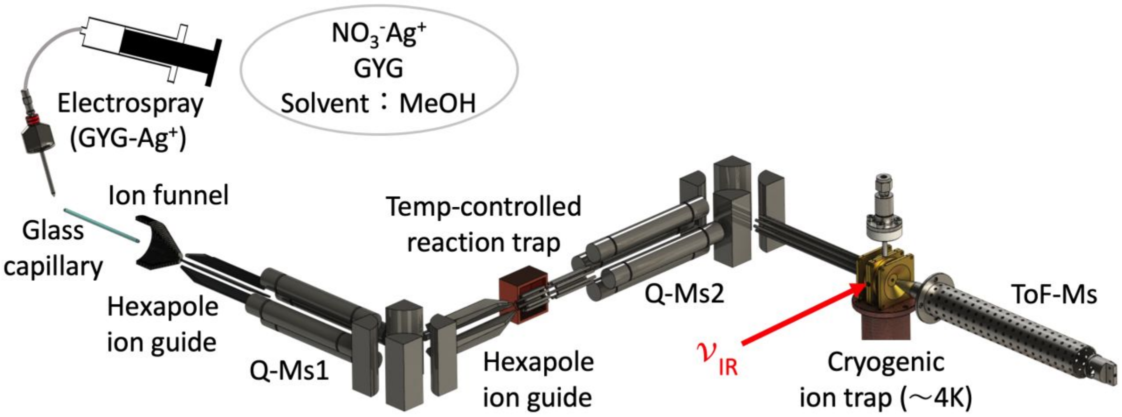

Fig. S7 Experimental setup for ion trap laser spectroscopy with the ESI source.

Natural Bond Orbital (NBO) calculation results for O/O/R structure

M^+…C=C interaction energy *E(1)* and *E(2)* for the Tyr-ring and the metal ion M^+ (K^+, Na^+ and Ag^+) in O/O/R conformer was determined by Natural Bond Orbital (NBO) analysis (Table S3 and S4). The value of *E(1)* and *E(2)* mean the interaction energy between donor NBO (i) and acceptor NBO (j), indicating the degree of interaction between donor and acceptor. Specifically, the interaction energy *E(1)* for σ-donation means degree of orbital interaction between the π orbital of C=C and the empty s-orbital of the metal ion. And, the interaction energy *E(2)* for π-back donation means degree of interaction between the p-orbital or d-orbital of the metal ion and the antibonding orbital π* of C=C. *E(1)* and *E(2)* are estimated by 2nd-order perturbation analysis as $E(n) = qi\ F(i,j) / E(j)\text{-}E(i)$. Where *qi* is the occupancy of the donor orbital. *E(i)* and *E(j)* are the orbital energies of the donor and acceptor NBO orbitals (diagonal elements). *F(i,j)* is the overlapping between the i and j NBO orbitals (off diagonal Fock matrix element or Kohn-Sham matrix element). For the Ag^+ complex, *E(1)* of σ-donation, C=C(π)→Ag^+(5s), was estimated to be 10.54 kcal/mol. And, *E(2)* of π-back donation, Ag^+(4d)→C=C(π*), was estimated to be 4.35 kcal/mol. In contrast, for the *E(1)* of σ-donation at Na^+ complex and K^+ complex, C=C(π)→Na^+(3s): 1.29 kcal/mol and C=C(π)→K^+(4s): 0.21 kcal/mol were estimated. And, for the *E(2)* of π-back donation at Na^+ complex and K^+ complex, Na^+(2p)→C=C(π*): 0.12 kcal/mol and K^+(3p)→C=C(π*): 0.07 kcal/mol were estimated. The *E(1)* and *E(2)* of the Ag^+ complex differ from those of other metal ion complex by at least a factor of 10, indicating difference is in order magnitude. This means that the interaction between Ag^+ and Tyr-ring is very strong, indicating that it is covalent (coordinate covalent bond).

Table S3. NBO analysis results for σ-donation C=C(π)→M^+(s)

Interaction	Donor NBO (i)	Occupancy (i)	Label of NBO (i)	Hybrization	Acceptor NBO (j)	Label of NBO (j)	Hybrization	*E(1)* kcal/mol	*E(j)-E(i)* a.u	*F(i,j)* a.u
C=Cπ → Ag^+5s	C_{23} - C_{24}	1.70277	BD	spC_{23} (99% 2p-char.)<br>spC_{24} (99% 2p-char.)	Ag_{34}	LP*	sp(99% 5s-char.)	10.54	0.38	0.059
C=Cπ → K^+4s	C_{23} - C_{24}	1.70740	BD	spC_{23} (99% 2p-char.)<br>spC_{24} (99% 2p-char.)	K_{34}	LP*	sp(97% 4s-char.)	0.21	0.32	0.008
C=Cπ → Na^+3s	C_{23} - C_{24}	1.71594	BD	spC_{23} (99% 2p-char.)<br>spC_{24} (99% 2p-char.)	Na_{34}	LP*	sp(99% 3s-char.)	1.29	0.34	0.02

Table S4. NBO analysis results for π-back donation M^+(p/d)→C=C(π)

Interaction	Donor NBO (i)	Occupancy (i)	Label of NBO (i)	Hybrization	Acceptor NBO (j)	Label of NBO (j)	Hybrization	*E(2)* kcal/mol	*E(j)-E(i)* a.u	*F(i,j)* a.u
Ag^+4d → C=Cπ*	Ag_{34}	1.97918	LP	d (99% 4d-char.)	C_{23} - C_{24}	BD*	spC_{23} (99% 2p-char.)<br>spC_{24} (99% 2p-char.)	4.35	0.34	0.038
K^+3p → C=Cπ*	K_{34}	1.99868	CR	p (100% 3p-char.)	C_{23} - C_{24}	BD*	spC_{23} (99% 2p-char.)<br>spC_{24} (99% 2p-char.)	0.07	0.78	0.007
Na^+2p → C=Cπ*	Na_{34}	1.99953	CR	p (100% 2p-char.)	C_{23} - C_{24}	BD*	spC_{23} (99% 2p-char.)<br>spC_{24} (99% 2p-char.)	0.12	1.17	0.012

The subscript numbers of the elements are the numbering in the coordinate data of optimized structure listed below (GYG-M^+(Na^+ and K^+) complex's C_{23} and C_{24} are the same Tyr-benzene-carbon moieties as those of GYG-Ag^+ listed below (Fig. S8)). The structure with labeled subscript numbers for the elements is shown in Fig.S8. For the NBO labels, BD, LP, and CR mean 2-center bond, 1-center valence lone pair, and 1-center core pair, respectively. Specifically, for the C_{23}-C_{24}, the donor NBO is the bonding orbital and the acceptor NBO is the antibonding orbital. For the metal ion, the acceptor NBO is the empty s-orbital occupied by the outermost electron. The donor NBOs of Ag^+, K^+, and Na^+ are the outermost electron orbitals 4d, 3p, and 2p of the ionic state.

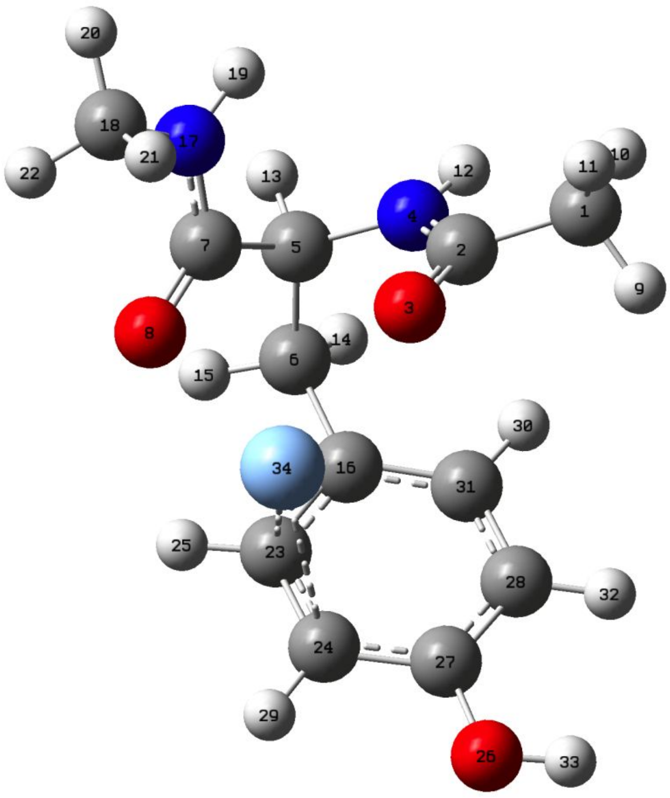

Fig. S8 Structure with labeled subscript numbers for the elements

Coordinate data for four optimized structures (O/O/R, O/R, O/O, O/O')

No.	OOR	x	y	z	OR	x	y	z
1	C	-0.974261	2.955076	1.936364	C	-0.925922	3.214791	1.478374
2	C	-1.080311	1.764395	1.020628	C	-0.821263	2.007264	0.590621
3	O	-0.774818	0.640125	1.428487	O	-0.067162	2.053962	-0.407308
4	N	-1.542668	2.002355	-0.229679	N	-1.565352	0.946174	0.913878
5	C	-1.673408	1.029398	-1.319328	C	-1.839416	-0.220936	0.086904
6	C	-0.414993	0.924538	-2.218782	C	-1.174559	-1.514764	0.631823
7	C	-2.195448	-0.328442	-0.794335	C	-3.371285	-0.418162	0.136114
8	O	-1.542801	-1.370090	-0.900062	O	-3.975201	-0.099635	1.148312
9	H	0.074058	3.084694	2.204848	H	0.071819	3.493525	1.814235
10	H	-1.350258	3.880631	1.505020	H	-1.308566	4.042124	0.880367
11	H	-1.518599	2.735771	2.853539	H	-1.574353	3.064818	2.338367
12	H	-1.738947	2.961206	-0.464540	H	-2.239733	1.044956	1.665048
13	H	-2.466924	1.418867	-1.960586	H	-1.495517	-0.014813	-0.925634
14	H	-0.339153	1.870712	-2.757513	H	-1.512735	-2.345048	0.009549
15	H	-0.618494	0.154287	-2.962056	H	-1.562843	-1.691809	1.634994
16	C	0.891136	0.646489	-1.521230	C	0.327182	-1.455439	0.651130
17	N	-3.417631	-0.297898	-0.256871	N	-3.925276	-1.000460	-0.941808
18	C	-4.033038	-1.474651	0.346070	C	-5.346618	-1.320771	-1.029853
19	H	-3.888773	0.587133	-0.171376	H	-3.352947	-1.159656	-1.753463
20	H	-5.101609	-1.304301	0.442024	H	-5.777837	-1.231936	-0.037540
21	H	-3.610016	-1.672224	1.332226	H	-5.859597	-0.632671	-1.701716
22	H	-3.859260	-2.339430	-0.288544	H	-5.476014	-2.339417	-1.391208
23	C	1.415841	-0.654623	-1.455018	C	1.034458	-1.148956	1.812145
24	C	2.569170	-0.937399	-0.709569	C	2.417670	-0.997204	1.815774
25	H	0.948020	-1.446258	-2.025042	H	0.495274	-1.022720	2.741462
26	O	4.363917	-0.230676	0.627710	O	4.481691	-1.024523	0.549784
27	C	3.252215	0.106600	-0.060209	C	3.143093	-1.139739	0.633996
28	C	2.775406	1.408711	-0.180066	C	2.460266	-1.454449	-0.562996
29	H	3.013164	-1.923440	-0.719212	H	2.935175	-0.777905	2.740862
30	H	1.265170	2.685068	-0.977539	H	0.564884	-1.998587	-1.422894
31	C	1.612968	1.663291	-0.901149	C	1.062739	-1.643568	-0.528432
32	H	3.315684	2.225269	0.281876	H	3.040940	-1.726511	-1.434508
33	H	4.793563	0.547594	0.999903	H	4.876054	-0.848957	1.412139
34	Ag	0.352392	-1.283659	0.680925	Ag	1.516972	0.715697	-1.084988

OO'	x	y	z	OO	x	y	z
C	-0.532109	-1.701431	2.632636	C	-0.941065	3.243546	1.242213
C	0.136743	-1.122238	1.418228	C	-1.231653	2.003224	0.443995
O	0.738062	-1.867187	0.629928	O	-2.399887	1.750991	0.107262
N	0.019094	0.211330	1.236813	N	-0.178555	1.228440	0.116219
C	0.239054	0.913719	-0.028560	C	-0.218267	0.072917	-0.776169
C	-0.938091	1.868289	-0.306159	C	1.025950	0.028231	-1.701315
C	1.638094	1.551642	-0.049944	C	-0.520041	-1.202731	0.031262
O	2.644576	0.871613	-0.329688	O	-1.685410	-1.647094	0.096493
H	0.117857	-2.451653	3.075911	H	-0.465107	-2.776545	2.288569
H	-0.795871	-0.952253	3.376635	H	1.212239	-3.258505	1.958514
H	-1.444194	-2.201829	2.302948	H	-0.105698	-3.809717	0.902074
H	-0.545634	0.700374	1.912912	H	0.727223	1.490193	0.473761
H	0.251459	0.159288	-0.814471	H	-1.081473	0.202377	-1.425868
H	-0.735999	2.387673	-1.244652	H	0.925389	0.877641	-2.378693
H	-0.993068	2.631086	0.476606	H	0.936783	-0.869365	-2.314092
C	-2.251517	1.130206	-0.369833	C	2.382128	0.074299	-1.038099
N	1.759621	2.840243	0.247679	N	0.470903	-1.796274	0.681577
C	3.055149	3.518334	0.267803	C	0.263735	-2.983398	1.507056
H	0.931162	3.382295	0.421572	H	1.407300	-1.436115	0.570571
H	2.907688	4.538016	0.610397	H	-1.241825	4.107379	0.649388
H	3.494505	3.528189	-0.728369	H	0.105941	3.346906	1.519567
H	3.735575	3.001242	0.940966	H	-1.558112	3.235426	2.139320
C	-3.171529	1.202439	0.672303	C	3.152683	-1.079191	-0.873038
C	-4.359602	0.482355	0.636572	C	4.394981	-1.040005	-0.252151
H	-2.977370	1.845739	1.522774	H	2.791210	-2.025282	-1.257074
O	-5.779377	-1.062293	-0.569208	O	6.101022	0.291724	0.830544
C	-4.643844	-0.330686	-0.458936	C	4.902993	0.172373	0.213992
C	-3.731785	-0.410072	-1.513905	C	4.163372	1.341839	0.027882
H	-5.067827	0.562018	1.451798	H	4.977149	-1.947185	-0.149153
H	-1.865810	0.251595	-2.296775	H	2.401902	2.214303	-0.784829
C	-2.554867	0.314258	-1.462927	C	2.929272	1.286515	-0.596584
H	-3.972023	-1.032882	-2.363513	H	4.585025	2.281393	0.355072
H	-6.359444	-0.910259	0.184332	H	6.557371	-0.555787	0.868060
Ag	2.611228	-1.379523	-0.546647	Ag	-3.450470	-0.250489	-0.141456

Reference

(1) Ishiuchi, S.; Sasaki, Y.; Lisy, J. M.; Fujii, M. Ion-Peptide Interactions between Alkali Metal Ions and a Termini-Protected Dipeptide: Modeling a Portion of the Selectivity Filter in K^+ Channels. *Phys. Chem. Chem. Phys.* **2019**, *21* (2), 561-571.

(2) Otsuka, R.; Hirata, K.; Sasaki, Y.; Lisy, J. M.; Ishiuchi, S.; Fujii, M. Alkali and Alkaline Earth Metal Ions Complexes with a Partial Peptide of the Selectivity Filter in K^+ Channels Studied by a Cold Ion Trap Infrared Spectroscopy. *ChemPhysChem* **2020**, *21* (8), 712-724.
